# Vaccination for COVID-19 among historically underserved Latino communities in the United States: Perspectives of community health workers

**DOI:** 10.3389/fpubh.2022.969370

**Published:** 2022-10-18

**Authors:** Luz M. Garcini, Arlynn M. Ambriz, Alejandro L. Vázquez, Cristina Abraham, Vyas Sarabu, Ciciya Abraham, Autumn K. Lucas-Marinelli, Sarah Lill, Joel Tsevat

**Affiliations:** ^1^Center for Research to Advance Community Health (ReACH), University of Texas Health Science Center at San Antonio, San Antonio, TX, United States; ^2^Department of Medicine, Joe R. and Teresa Lozano Long School of Medicine, University of Texas Health Science Center at San Antonio, San Antonio, TX, United States; ^3^Department of Psychiatry and Behavioral Sciences, Joe R. and Teresa Lozano Long School of Medicine, University of Texas Health Science Center at San Antonio, San Antonio, TX, United States; ^4^Baker Institute Center for the United States and Mexico, Rice University, Houston, TX, United States; ^5^Department of Psychology, Utah State University, Logan, UT, United States; ^6^Department of Sciences, University of Texas at San Antonio, San Antonio, TX, United States; ^7^Department of Psychology, Trinity University, San Antonio, TX, United States; ^8^Department of Population Health and Internal Medicine, Dell Medical School, University of Texas, Austin, TX, United States

**Keywords:** vaccine, COVID-19, Latinos/Hispanics, community, health disparities, hesitancy

## Abstract

A critical step to reduce the spread of COVID-19 is vaccination. We conducted a mixed methods project that used online surveys and focus groups with 64 Community Health Workers and *Promotor/as* (CHW/Ps) located near the U.S.-Mexico border to identify barriers and facilitators to COVID-19 vaccination among Latino communities that have been historically underrepresented and medically underserved. Overall, personal barriers to vaccination included mistrust of manufacturers and administrators as well as fear of: becoming infected from the vaccine, discrimination/stigmatization from healthcare professionals administering the vaccine, exploitation/manipulation by the government or health authorities, and having personal information mishandled. Environmental and community barriers included being undocumented and fear-inducing myths and beliefs. Additional barriers included limited information and logistics pertaining to vaccination access. Targeted efforts are needed to overcome barriers in a culturally and contextually sensitive manner to prevent harm and reduce risk of infection among communities that have been historically underrepresented.

## Introduction

The COVID-19 pandemic has disproportionately affected ethnic/racial underrepresented communities and has been particularly devastating for many Latino communities in the United States (U.S.) (1). According to the Centers for Disease Control and Prevention (CDC), Latinos in the U.S. are 1.5 times more likely to be infected with COVID-19 and 2.3 times more likely to be hospitalized for COVID-19 when compared to non-Latino Whites (2). In Texas, the U.S. state with the second largest Latino population (39.3% of the state's population) (3), Latinos represented 26.43% of all confirmed COVID-19 cases compared to 22.37% of Whites as of May 14, 2022 (4). Equally of concern is that when compared to non-Latino Whites, Latinos in the U.S. are 1.8 times more likely to die from COVID-19 (2). Latinos make up 583.2 of age-adjusted COVID-19 fatalities per 100,000 individuals in Texas, in comparison with only 309.0 fatalities per 100,000 for non-Latino Whites as of April 23, 2022 (5). The toll of COVID-19 among Latinos in the U.S. is also evident among younger Latinos, particularly those of working age (6). Indeed, estimates suggest that the Latino health advantage in all-cause mortality rates (a phenomenon known as the Latino paradox) is falling by over 70% due to COVID-19, a phenomenon that may persist beyond the current pandemic due to its related long-term health, social, and economic impacts (6).

An important way to control and end the COVID-19 pandemic is vaccination (7). To this end, equitable and sustainable vaccine administration requires identifying barriers to access and factors contributing to vaccine hesitancy among high risk groups, such as Latinos (8). According to the Kaiser Family Foundation, the current rate of at least one dose of the COVID-19 vaccine among Latinos in Texas is 65%, as of April 4, 2022, which is somewhat higher than the 54% vaccination rate among non-Latino White Americans and markedly higher than the 48% vaccination rate among African Americans (9). Asian Americans, meanwhile, have the highest vaccination rate at 75% (9). Barriers and facilitators to vaccination may differ across settings and populations depending on the contextual environment, available resources, and prevalent cultural myths, beliefs, and practices (10, 11).

Our current understanding of barriers to vaccination among Latino populations suggests four interconnected categories: (a) vaccine hesitancy among individuals and within communities, (b) inaccessibility of vaccine information, (c) structural issues relating to distribution and accessibility; and (d) concerns specific to certain groups within Latino communities, such as those of immigrant status (12–15). Many of the aforesaid barriers are rooted systemic inequalities in the healthcare system and cultural variations that disproportionately affect the health outcomes of Latino individuals (12).

Personal beliefs and concerns surrounding vaccination among Latinos often result in vaccine hesitancy (13), which prior research has attributed to historical mistreatment of racial and ethnic minority populations leading to lasting mistrust of the healthcare system (12), perceptions of limited vaccination supply in Latino communities (12), and vaccine misinformation (13). These aforementioned deterring beliefs often cluster within social networks and maintain myths that interfere with vaccination efforts within Latino communities (14). Coupled with widespread vaccine misinformation (14), myths and historically-rooted mistrust in the healthcare field work synergistically to foster challenges for widespread and equitable immunization (14).

Importantly, certain subpopulations within Latino communities, such as those with undocumented immigration legal status, those of lower socioeconomic status, and those without medical insurance, also face unique barriers to vaccine access (15). For example, Hamel et al. (16) found that over half of vaccinated Latino individuals reported being asked for government-issued identification prior to vaccination, and many unvaccinated Latinos expressed concerns with being asked to provide documentation (15). While immigration status does not preclude vaccination, having to present identification may pose a key obstacle to those of immigrant status (15). Additionally, Latinos of lower socioeconomic status may be deterred by their employers' lack of paid time off to recover from potential side effects of vaccination (15). These group-specific obstacles are important to consider given their ability to significantly limit vaccine participation. As the landscape of COVID-19 vaccination and the need for booster sessions continues to evolve, and in light of the disparate impact COVID-19 has had on Latino populations, a greater understanding of the barriers and facilitators to vaccination in underserved Latino communities is critical.

## Purpose

This project was guided by principles of community-engaged research through collaboration with community health workers (CHWs) and *Promotor/as* (CHW/Ps) who serve the needs of communities that are medically underserved in South Texas, near the U.S.-Mexico border. *Promotor/as* are trained Latino community members who provide health education and resources to Latino communities. CHWs have played a key role in providing health information to Latino communities over the course of the COVID-19 pandemic, and are essential to support continued efforts to achieve health equity. Using the perspectives of CHW/Ps, the goal of this project was to identify cultural and contextual barriers and facilitators to vaccination for COVID-19 among Latino communities that have been historically underrepresented and/or that have been medically underserved, in particular those in remote or rural locations in proximity to the U.S.-Mexico border.

## Methods

From its inception, the development of this project relied on shared cultural and contextual knowledge from collaborating CHW organizations, including two CHWs that were included as essential members of the research team. The collaborative process with local and regional CHW organizations began 3 months prior to the launching of this project; this time was used by the principal investigator to identify and learn about the priorities and needs of CHWs and their communities in the targeted region. In the process, the principal investigator along with collaborating CHWs visited community organizations and provided online psychoeducational webinars on self-care for local/regional CHWs. This provided an opportunity for CHWs in the region to become familiar with our work, while our team listened to their needs.

To facilitate obtaining diverse data, we recruited bilingual CHW/Ps using network-based referrals from the collaborating CHW organizations in communities that have been historically underrepresented and medically underserved in the region near the U.S.-Mexico border. Our collaborating CHW associations have an extensive history of facilitating outreach, research and the provision of various health services to Latino communities that have been historically marginalized. Specific strategies used in the recruitment included emails, social networks, and recruitment throughout online meetings for the local CHWs' associations. Eligibility criteria required that CHWs and/or *Promotor/as* be fluent in English and/or Spanish, and that they resided and worked with Latino communities that have been historically underserved.

We collected data from a sample of 64 CHW/Ps by using mixed methods, specifically qualitative and quantitative data (17). All data were collected remotely to avert person-to-person exposure during the pandemic. We collected quantitative data *via* an online Qualtrics survey (18), and qualitative data was collected using online focus groups *via* Zoom that were facilitated by a bilingual facilitator and two bilingual research assistants, including one of the two collaborating CHWs that were members of our research team. Surveys and focus group questions were first prepared in English, translated into Spanish, and then back-translated into English for consistency. The final survey and focus group questions were pre-tested in a small group of CHWs. A final version of the survey and focus group questions was established following feedback from the pre-test. All participants completed the online survey prior to attending a focus group. This strategy was helpful to introduce the topic to the participants and motivate in-depth discussion during the focus groups. Out of the five focus groups conducted, two were in done in English and three in Spanish. The online focus groups and the online survey each took about an hour to complete. Participants who completed the survey and participated in a focus group received $40 as compensation. Participants consented to audiotaping the focus groups, and all audiotapes were transcribed to facilitate data analysis. The *blinded* Institutional Review Board deemed this project non-regulated research on the basis of the project being a needs assessment project (HSC20200349N).

### Measures

In line with community-engaged research, the survey and focus group questions were developed in collaboration with community partners from local CHW associations. The online survey consisted of 80 questions and included structured and open-ended questions. The survey included questions to assess: (a) demographic information (i.e., gender, age, race, were used. ethnicity, and education); (b) working expertise and history as CHW (i.e., work status, work setting, credential/licensing information, years working as CHW, demographic characteristics of their clients/patients, types of communities where their work takes place, and financial support for their work); and (c) perceived willingness of people in their communities to engage in COVID-19 vaccination; and (d) barriers and facilitators to COVID-19 prevention efforts, including vaccination for COVID-19. To assess barriers to COVID-19 vaccination, we asked, “On a scale from 1 (not at all) to 4 (extremely), how likely is it that the following list of concerns may prevent people in your community from getting vaccinated for COVID-19?” Responses were provided using a Likert scale ranging from no concern or barrier to serious concern or barrier. Open-ended questions were used to assess for any domains that may have not been included in the lists of barriers and facilitators to COVID-19 vaccination.

For the qualitative data, a topic guide was developed in collaboration with local CHW associations, which was used to guide the discussions for the focus groups. Questions for the focus groups were semi-structured and were aimed at fostering discussion pertaining to: (a) beliefs, attitudes, and barriers to vaccination for COVID-19 among Latino communities that have been historically underrepresented and/or medically underserved, and (b) facilitators to increase knowledge about and willingness to get the vaccine. As recommended by our collaborating CHWs, at the end of each interview or group, we provided participants with an opportunity to provide us with anonymous feedback using a comments and suggestion sheet. This was extremely helpful and provided us with knowledge that we incorporated into subsequent groups (e.g., provide information about resources for local mental health and legal services; explain the importance of research; schedule future talks in the community; longer duration of the groups to allow for more in-depth discussion).

## Analyses

Quantitative data were analyzed using SPSS software. Descriptive statistics were used to develop a demographic profile of participating CHWs and the communities they serve, as well as to analyze data on barriers and facilitators to COVID-19 vaccination. Over a 2-month period, weekly group meetings with the principal investigator, research assistants, and collaborating CHWs were scheduled to analyze the findings from the focus groups. Qualitative data were analyzed through systematic methods outlined by Miles and Huberman (13), including starting with specific questions previously developed and then proceeding through the steps of data categorization, data reduction, data display, and conclusion drawing and confirmation (19). To confirm the categorization and validity of the data, we triangulated the data by comparing survey data with data from the focus groups and notes from all members of our research team. Upon completion of data analyses, the results described below were presented and discussed with our collaborating CHW organizations, and modifications were made with the feedback provided.

## Results

### Participants

Overall 64 CHW/Ps participated in this project. Most CHW/Ps were women of Latino background. Their mean age was 45.7 (*SD* = 13.3). Most (84.4%) reported working with low-income communities; 46.9% regularly worked with immigrant communities; and most worked across various settings, including community locations (e.g., homes, schools, community organizations; 62.5%), non-profit offices or facilities (50.0%), churches or faith-based organizations (21.9%), and primary care clinics (14.1%) (see Table 1).

**Table 1:** Demographic characteristics of community health workers and *Promotor/as*.

**Characteristics**	**Total (*N* = 64)**
**Sex**	
% Women	89.8
Age, years (*M, SD*)	45.3 (13.7)
**Race/ethnicity**	
% Latino	91.5
**Education**	
% Graduating high school	89.8
**Employment**	
% Employed full or part time	85.7
Years worked as a CHW/P *(M, SD)*	6.4 (7.0)

### Perceived relevance and importance of vaccination

Overall, using a scale from 1 (strongly disagree) to 5 (completely agree), 82.5% of CHW/Ps agreed or completely agreed that vaccination can provide important benefits to society. In addition, 77.2% of CHW/Ps agreed or completely agreed that people in their communities perceive vaccines in general as useful and effective. Yet, a substantial proportion of CHW/Ps (43.8%) agreed or completely agreed that people in their communities are likely to think of vaccines as having the potential for harmful side-effects. Indeed, 28.1% of CHW/Ps agreed or completely agreed that vaccines may sometimes cause illness or death. Nevertheless, most CHW/Ps (70.7%) reported that people in their communities would likely or definitely get vaccinated for COVID-19 if the vaccine were available to them. Of note, 8.6% of participants reported that people in their communities would be unlikely or extremely unlikely to get vaccinated for COVID-19, with 20.7% of participants reporting being unsure.

### Barriers to vaccination for COVID-19 among underserved Latino communities

#### Personal fears or concerns

Survey results showed that the most frequently reported barriers to vaccination at the personal level were fear and mistrust, as well as concerns related to the COVID-19 vaccine itself. For instance, fear of getting exposed to COVID-19 from others at the vaccination site was identified as a salient concern (4-point Liker scale *M* = 2.39; *SD* = 0.71), followed by fear of contagion from the vaccine itself (*M* = 2.33; *SD* = 0.71), mistrust of vaccine manufacturers and distributors (*M* = 2.34; *SD* = 0.71); concerns about being discriminated by healthcare professionals administering the vaccine (*M* = 2.04; *SD* = 0.77); and worry about personal information being mishandled (*M* = 1.80; *SD* = 0.79).

In addition to the aforementioned fears and concerns, results from qualitative data identified exploitation and manipulation from government and health authorities as salient concerns associated with ethnic/racial social injustices and health inequities, as well as mistrust from the government in mishandling personal information. Another barrier identified pertained to the novelty of the COVID-19 vaccine: CHW/Ps emphasized that the unprecedented nature of COVID-19 along with limiting and confusing information surrounding the virus has magnified existing fears about COVID-19 vaccination. Moreover, CHW/Ps reported that some community members mistrust vaccine manufacturers for developing the vaccine without apparent sufficient time to undergo extensive safety testing, and mistrust healthcare workers administering the vaccine for perceived inadequate expertise or reputation for delivering vaccines. Some in their communities worried about vaccines being administered in hospital settings where the person going to get vaccinated could catch COVID-19 or other infectious diseases (Table 2).

**Table 2:** Personal, environmental, and community barriers to vaccination for COVID-19 according to CHW/Ps.

**Barriers**	**Sub-categories**	**Sample quotations**
Fears/concerns	1. Becoming infected from others	[People] worry that they are going to be sick … I think that going around other people that might have [the virus] gives them concern … making contact or getting exposed [at vaccination site].
	2. Getting COVID-19 from the vaccine itself	Many people have told me “I am not getting the vaccine because they will be injecting us with the virus” … they are afraid that if they get the shot, they will acquire the virus. People are fearful that they will get sick.
	3. Exploitation or manipulation	I know that the community of Laredo is very afraid because they were chosen to be a testing site for the vaccine. [People] are afraid that they are being racially chosen [to experiment] because a big majority of it is Hispanic and also it's a very poor area … a lot of people in Laredo are very afraid.
Mistrust	1. Novelty	[People] are afraid because the vaccine is new and might not work properly or [may] affect them negatively … new things are coming and other things will appear with the vaccine … I think twice before getting any shot or getting my kids vaccinated. I think that [people] are already afraid of the vaccines that already existed so when there is a new vaccine and [people] don't know about it, people are going to get even more fearful.
	2. Mishandling personal information	Fear that [the vaccine] is a lie … it has to do with the government … it has to do with the information the government receives from people.
	3. Of manufacturers of vaccine	[People feel] that the vaccine was rushed without measuring side effects. [People] fear the vaccine will make them more sick because they did not develop it with enough time and testing as [is done] with other diseases … who will be willing to get vaccinated, if it hasn't even been a year?
	4. Of administrators of vaccine	I think that places where people can see that they are not experienced doing [vaccinations] might inspire doubt or fear of whether they know what they are doing … if these are places where they normally give all the other vaccines, then [people] feel that they know what they're doing. In certain communities, there's a general belief that hospital make you more sick … they don't know what they are doing … if you go there, they're probably going to give you more diseases or more infection, and you don't know what kind of people are coming there. So that's another fear, that you may go for a vaccine, but you will bring back more diseases … there are many doctors who don't know where they're going.
Myths/beliefs	1. Myths (fear-inducing)	One of the [myths] going around is that if you get the shot, they would be placing a chip with it, so then [the government] would have more control over us. [The government] is implanting a chip—nanotechnology—by using the vaccine, so that would lead to control … [people] are not having the trust.
	2. Myths (coercion)	Someone told me that they had been told they were going to force all the children to get vaccinated before going to school … when this arises and these fears spread, it does affect [the community]. A lot of people are saying that they regret getting tested for COVID, because those [people] will get the vaccine, whether they want to or not, because they had the virus, already.
	3. Religious beliefs	[People] involve religion a lot … they have certain beliefs … there is talk about [the vaccine] being the mark … it's in the Bible … it talks about the apocalypse and the mark of the beast that people are going to be marked with … marked with the vaccine … It's not exactly the fear itself to the vaccine, but the fear to be doing something wrong on their faith or against their God. Always based on our beliefs, whether religious or whatnot, this stigma is present.
	4. Folk/natural healing practices	My son-in-law prefers organic medications and natural products so my grandchildren have not been vaccinated … he does not believe in vaccines … when my grandchildren were born, the midwives did waterbirths right there, because he did not want them to be born at a hospital and get vaccinated.
Environmental factors	1. Immigration legal status	If the people don't have documents [or are undocumented], it is hard for them to get vaccinated … not knowing what the criteria is, whether or not they'll accept their *Matrícula*, whether or not they'll accept their identification, if they have an identification or if it's expired … there is a risk.
	1. Limited information	There is a lot of information missing … what it's not true or what is true, right? Is medical insurance required? When there is no information available, [people] start making up a lot of myths about the vaccine.

#### Environmental and community level factors

Survey results showed that a salient barrier at the environmental and community level was confusing information about the COVID-19 vaccines *(M* = 2.42; *SD* = 0.69). Difficulties in getting access to a vaccination site (e.g., due to lack of transportation and conflicting work schedules) *(M* = 2.36; *SD* = 0.73) was also identified as a barrier.

Qualitative data identified immigration legal status as interfering with willingness to get vaccinated. For instance, participants said that people with undocumented immigration legal status are concerned about not having the required documentation (e.g., driver license and other form of identification) to receive the vaccine and that they feared that disclosing personal information could result in detention and/or deportation. Also, certain myths and beliefs could interfere with vaccination, among them myths or religious beliefs precipitating fear, worry, shame, and guilt. For instance, one fear-inducing myths was that vaccination will be used as a surveillance strategy by the government to control the population and to manipulate information. Another myth was that vaccination was a coercive strategy to reduce people's sense of agency. A prevailing religious belief was that vaccination *per se* may go against God's will or fate, thus leading to a punishable sin. CHW/Ps also highlighted the preference for folk or traditional healing practices, such as herbal or natural remedies, instead of vaccination to fight off diseases.

#### Vaccine fears or concerns

A prevalent fear or concern related to the vaccine itself was the potential cost of the vaccine (*M* = 2.65; *SD* = 0.62), given that some people do not know that the vaccine is free and that they are concerned about possible money scams. Also, concerns about the safety of the vaccine, such as possible side effects (*M* = 2.37; *SD* = 0.68) and efficacy of the vaccine (*M* = 2.34; *SD* = 0.65), were reported as vaccine-related concerns. Results from qualitative data further identified concerns about vaccine efficacy, specifically a belief that people may contract the virus when exposed to others even if they have been vaccinated (see Table 3).

**Table 3:** Vaccine-related concerns as barriers to vaccination for COVID-19 according to community health workers/*Promotor/as*.

**Sub-categories**	**Sample quotations**
1. Cost/affordability	My concern is that only the affluent and only the people that have access to healthcare with insurance are getting [the vaccine.]
2. Effectiveness	The knowledge we have so far [about the vaccine] … it's not enough and [people] are afraid. I want to observe them, see how [people] react [to vaccination] … see when they are in contact with others … Yes, it's going to be the same thing over again, and we are going to have to wait some time for people to gain trust.
3. Side effects	I've done a small survey with a few [people] … they say they would not [get vaccinated] … they would wait because they don't know what side effects or what could happen … typically when you get the shot, there is a reaction, and what reaction will it be? [People] are not going to get the vaccine until there are enough studies that show the results … there will be people that want to see the facts.

### Facilitators to COVID-19 vaccination in underserved Latino communities

#### Knowledge about the COVID-19 vaccine

Survey results identified specific knowledge domains about the COVID-19 vaccine important to address among Latino communities that are medically underserved. First, any information about the vaccine needs to be available in English and Spanish (*M* = 2.86; *SD* = 0.35). Specific topics should include: (a) vaccine effectiveness (*M* = 2.82; *SD* = 0.49); (b) possible side effects (*M* = 2.80; *SD* = 0.44); (c) how the vaccine is administered (*M* = 2.79; *SD* = 0.49); (d) how long protection from the vaccine lasts (*M* = 2.75; *SD* = 0.51); (e) available vaccination sites and schedules (*M* = 2.72; *SD* = 0.53); (f) that vaccination is free of charge (*M* = 2.71; *SD* = 0.56); and (g) who will have access to vaccination records and related personal information (*M* = 2.62; *SD* = 0.59).

#### Environmental and community facilitators to vaccination for COVID-19

In addition to the need for information about the vaccine, quantitative results showed that the availability of bilingual staff to administer the vaccine is important (*M* = 2.91; *SD* = 0.29), as well as ensuring that there is ample supply of vaccines (*M* = 2.86; *SD* = 0.40). Moreover, convenient locations (*M* = 2.84; *SD* = 0.37), convenient vaccination schedules (*M* = 2.64; *SD* = 0.75), short wait times (*M* = 2.72; *SD* = 0.59), an option to get vaccinated at home (*M* = 2.37; *SD* = 0.87), and confidentiality are key (*M* = 2.47; *SD* = 0.89).

Findings from qualitative data emphasized that trusted networks are essential to delivering vaccine information, whether by building alliances with community partners or through testimonials from people in the community. In this regard, a CHW commented,

“We have to see that [the vaccine] worked with our own eyes because we can't just believe certain things when a lot of the stuff that's being said is here and there.”

The attitudes and personal attributes of people providing information and vaccinations, including medical providers, were cited as important. For instance, a CHW stated,

“Who are [people] going to trust? [People] are going to trust the worker that is genuine and compassionate. If they are not like that, patients don't trust… if [the provider] is abrupt or [if the provider] treats [people] like they're lower or that their education is low and they don't comprehend, then that's not a person that [people] are going to trust… genuine and compassionate is the name of the game.”

## Discussion

Vaccination for COVID-19 is paramount to preventing the continued spread of the disease, yet many barriers continue to interfere with vaccination efforts among Latino communities that are marginalized or medically underserved (19). Research on COVID-19 has focused primarily on identifying disparities related to the pandemic (e.g., infection, death, and vaccination rates) and relevant demographic factors, with less consideration given to personal and community factors that may influence such disparities. This study identified personal, community and environmental barriers to vaccination for COVID-19 among Latino communities that have been historically underrepresented and/or medically underserved, as well as important facilitators to consider in order to propel vaccination efforts.

Mistrust and fear among Latino communities have been prevalent for years, yet this has escalated significantly over the past 5 years as a result of harmful rhetoric, stigmatization of Latino immigrants, discriminatory practices, and punitive governmental actions (20, 21). Consistent with this history, our findings showed that mistrust and fear of the government and of healthcare infrastructure were endorsed as significant personal level barriers to COVID-19 vaccination (e.g., fear that personal information may be mishandled; concerns about poor and/or differential treatment, including poor follow-up care if side-effects or symptoms develop; skepticism toward vaccine manufactures, etc.). Our findings also show that fears of exploitation and manipulation, such as concern that people are being experimented upon to test the vaccine, are also important barriers to COVID-19 vaccination and need to be addressed. Noteworthy is that mistrust and fear are especially common among Latino immigrants with undocumented immigration legal status, their family members with documented status, and people living in communities with a large proportion of immigrants with undocumented or temporary immigration legal status. People in those communities are concerned that not having proper identification or documentation (e.g., driver license, government or state issued identification) may prevent them from being eligible for vaccination, and that even if people have some form of identification, they may be at increased risk for detention, deportation, and/or family separation if they are “caught” being vaccinated. All of the aforementioned concerns are fuelling fear-inducing myths (e.g., vaccination is a tool used by the government as a surveillance strategy), myths that spread rapidly in the community leading some people to avoid vaccination. Future research is needed to identify strategies for building trust and reducing fear among historically marginalized Latino communities, including informing ways to deliver health messages in a manner that is contextually and culturally appropriate.

Adequate information about COVID-19 and COVID-19 vaccines is crucial to promoting community health, increasing vaccination rates, and countering fear-inducing myths and beliefs. Unfortunately, the spread of misinformation about COVID-19 and COVID-19 vaccines has caused confusion and adversely affected vaccination efforts among Latinos (22). Our findings corroborate that the dearth of factual information, especially in Spanish and in online or digital sources has undermined vaccination efforts among Latinos, particularly those that are marginalized or those living in remote or rural areas that are medically underserved. It is important to make information available about how different types of vaccines work and how they are made, including how new vaccine technology (e.g., mRNA) has been applied successfully in cancer treatments and other vaccines (e.g., Zika, rabies, etc.) (23, 24). Use of simple language and visual aids can be helpful to overcome concerns about the novelty of the vaccine and fears about experimentation (25). Future studies focusing on the use of an interdisciplinary approach that brings diverse perspectives from marketing, healthcare and the social sciences to identify best strategies for delivering health messages to underserved Latino communities are needed to fill existing knowledge gaps and to inform health equity efforts.

To reduce fear and increase trust, health providers and public health authorities should partner with non-traditional sources of service delivery in communities that have been historically underserved. In this regard, community non-profits and faith-based institutions can be key collaborators (26). Likewise, CHW/Ps are trusted members of the community who work in tandem with local healthcare, community, and government systems as an extension for providing services (27). CHW/Ps play a variety of roles in education and healthcare delivery, including acting as liaisons with their communities, assisting with health system navigation and case management, counseling, combating misinformation and myths, and collecting data for disease surveillance, among many other important activities (16, 27). CHW/Ps are not only trusted by members of their communities, but they also understand cultural and contextual factors and interpersonal dynamics important to address to increase vaccination rates among Latino communities that have been historically marginalized (27). CHW/Ps can inform and deliver vaccination messages and materials that appeal to Latino cultural values, such as that vaccination is an act of love and care toward one's family and community, while also leveraging testimonials from community members attesting to the benefits of vaccination. To maximize success, collaborations between public health authorities and non-traditional sources of service delivery have to be carefully and respectfully established, adequately funded, and provided with ongoing training, support, and resources during and beyond the pandemic.

## Limitations

This study has several limitations. Our sample size was modest, and although drawn from a large region, represents Latino communities in South Texas that have been historically underrepresented and/or medically underserved. Latinos in the U.S. are a heterogeneous population, so a larger study to identify barriers and facilitators that may influence vaccination across different U.S. Latino communities is needed. Additionally, due to COVID-19, we conducted our survey and focus groups over a video conferencing platform (web-based and telephone-accessed), which may have excluded participants without access to the internet or reliable phone service. Nonetheless, we gathered diverse perspectives from a broad region in a manner that facilitated participation while complying with social distancing recommendations.

Also, data were collected in the summer of 2020, several months before COVID-19 vaccines first became available in the U.S. Future studies should compare results from this project with current trends to assess changes over time in barriers and facilitators to COVID-19 vaccination among historically marginalized Latino communities. This information is needed to inform continued prevention efforts and emergency preparedness for future events.

## Conclusion

Vaccinations play an essential role in maintaining the health of our communities. Vaccination for COVID-19 among Latino communities that have been historically marginalized is multifaceted, however. Targeted efforts that build upon collaboration with non-traditional sources of service delivery that are trusted by communities that have been underrepresented are needed to overcome barriers in a culturally and contextually sensitive manner. Not all of the barriers identified in this study are unique to COVID-19 vaccination efforts, but they highlight problems in the availability and accessibility of healthcare more broadly among Latino communities that have been historically marginalized. Advocacy and policy efforts aimed at increasing access to vaccination and preventive efforts to promote and maintain good health for this vulnerable population should be prioritized.

## Data availability statement

The raw data supporting the conclusions of this article will be made available by the authors, without undue reservation.

## Ethics statement

This project was reviewed and approved by UTHSCSA Institutional Review Board and was deemed non-regulated research on the basis of the project being a needs assessment project (HSC20200349N). The patients/participants provided their written informed consent to participate in this study.

## Author contributions

The first draft of the manuscript was written by LG and AA. All authors contributed to the study conception and design, material preparation, analysis, commented and edited on previous versions of the manuscript, read, and approved the final manuscript.

## Funding

This study was funded by a grant from the National Institutes of Health, National Heart, Lung, and Blood Institute (NHLBI) (K01HL150247; PI: LG) and was also supported with funds from the Joaquin G. Cigarroa, Jr. M. D. Distinguished Chair at the University of Texas Health Science Center at San Antonio.

## Conflict of interest

The authors declare that the research was conducted in the absence of any commercial or financial relationships that could be construed as a potential conflict of interest.

## Publisher's note

All claims expressed in this article are solely those of the authors and do not necessarily represent those of their affiliated organizations, or those of the publisher, the editors and the reviewers. Any product that may be evaluated in this article, or claim that may be made by its manufacturer, is not guaranteed or endorsed by the publisher.

## Author disclaimer

The content is solely the responsibility of the authors and does not necessarily represent the official views of the National Institutes of Health.
